# Successful control of a neonatal outbreak caused mainly by ST20 multidrug-resistant SHV-5-producing *Klebsiella pneumoniae*, Greece

**DOI:** 10.1186/1471-2431-14-105

**Published:** 2014-04-17

**Authors:** Angeliki Mavroidi, Apostolos Liakopoulos, Antonios Gounaris, Maria Goudesidou, Katerina Gaitana, Vivi Miriagou, Efthymia Petinaki

**Affiliations:** 1 Department of Microbiology, University Hospital of Larissa, Larissa, Greece; 2 Neonatal Intensive Unit, University Hospital of Larissa, Larissa, Greece; 3 Laboratory of Bacteriology, Hellenic Pasteur Institute, Athens, Greece; 4 Department of Microbiology, Medical School, University of Thessaly, Biopolis, Larissa, Greece

**Keywords:** Outbreak, *Klebsiella pneumoniae*, ESBL, NICU, Molecular typing

## Abstract

**Background:**

Extended spectrum beta-lactamase-producing *Klebsiella pneumoniae* (ESBL-Kp) infection can cause significant morbidity and mortality in neonates. We investigated a nosocomial ESBL-Kp outbreak in a neonatal intensive care unit (NICU) of the University Hospital of Larissa (UHL), Central Greece.

**Methods:**

A total of sixty-four ESBL-Kp were studied; twenty six isolates were recovered from the NICU and were compared with thirty-eight randomly selected isolates from different wards of the hospital during the period March- December 2012. All isolates were characterized by antimicrobial susceptibility testing, ESBL-production by double-disk synergy test, molecular typing using BOX-PCR, whereas selected isolates were further characterized by beta lactamase and virulence gene content, multilocus sequence typing and phylogenetic analysis. All neonates affected by ESBL-Kp were put under strict contact isolation, along with appropriate infection control measures.

**Results:**

The outbreak strain of ST20 multidrug-resistant SHV-5-producing *K. pneumoniae* was identified in all infected (n = 13) and three colonized neonates. A novel ST (ST1114) was also identified among SHV-5 producers (n = 10) recovered from nine colonized infants, but it was not related with ST20. Both STs were identified only in the NICU and not in other wards of the hospital. No ESBL-Kp were isolated from the hands of the nursing staff and the environment. Although we were not able to identify the source of the outbreak, no ESBL-Kp were isolated in the NICU after this period and we assumed that the outbreak was successfully controlled. All neonates received parenteral nutrition and most of them were delivered by caesarean section and showed low gestational age (<32 weeks) and low birth weights (<1500 g).

**Conclusion:**

According to our knowledge, this is the first description of an outbreak of multidrug-resistant SHV-5 producing *K. pneumoniae* assigned to ST20.

## Background

*Klebsiella pneumoniae* is an opportunistic pathogen responsible for nosocomial infections. The microorganisms are isolated more frequently from the stool, umbilical cord and the oropharynx. Bloodstream infections caused by *K. pneumoniae* are also often reported in the neonatal intensive care units (NICUs) [1]. Transmission can occur either from the mother to child at birth, or acquired during nursery by person-to-person transmission, via the hands of the nursing staff and the contaminated equipment, food or the environment.

In *K. pneumoniae*, acquired resistance to penicillins, broad spectrum cephalosporins and monobactams, except for carbapenems and cephamycins can be mediated by the production of extended-spectrum beta-lactamases (ESBLs). The most widespread ESBLs belong to the TEM, SHV and CTX-M families [2-5]. Choice of antibiotic therapy may be limited if the organism produces an ESBL, particularly for pneumonias. Nosocomial outbreaks of extended-spectrum beta-lactamase-producing *K. pneumoniae* (ESBL-Kp) with increased morbidity and mortality have been frequently reported, mostly in debilitated, hospitalized patients in the intensive care units (ICUs) and neonatal units [2-11].

During 2012, the emergence and spread of ESBL-Kp was documented in the neonatal intensive care unit (NICU) of the University Hospital of Larissa (UHL), Central Greece. We report here, the epidemiological features, molecular characterization of the beta-lactamase and virulence gene content, molecular epidemiology by BOX-PCR and multilocus sequence typing (MLST), and control measures for the outbreak of multidrug-resistant SHV-5 producers.

## Methods

### Setting and definition of cases

The UHL serves as one of the main (600-beds) tertiary care hospitals in the district of Thessaly, (1,000,000 inhabitants). The NICU of UHL receives approximately 750 admissions per year and it has six rooms (40 beds) for newborns of age less than or equal to 28 days. Outbreak cases were defined by isolation of an ESBL-Kp strain from any culture of infected neonates in the NICU. Infection was defined by clinical and laboratory criteria and requirement for antimicrobial therapy, while, colonization by the absence of relevant symptoms. We note that no routine screening for ESBL-Kp has been performed in the NICU before the onset of the outbreak because infections caused by these microorganisms were very rare in the NICU (Petinaki, unpublished data).

### Identification of isolates and antimicrobial susceptibility testing

Identification to the species level and antimicrobial susceptibility testing of the isolates has been performed by the Vitek-2 Advanced Expert system (BioMerieux Inc., Marcy l’ Etoile, France), according to the interpretive criteria of the Clinical and Laboratory Standards Institute- CLSI [12]. Phenotypic screening for ESBL production was performed by the double-disk synergy test (DDST) and for carbapenemase production by the meropenem-boronate combined disk test, as described previously [3,13].

### Bacterial isolates and surveillance cultures

Overall, a total of 64 non-carbapenemase-producing ESBL-Kp collected from March to December 2012 in UHL were analyzed; 26 of them were consecutively recovered from various clinical specimens in the NICU, whereas the remaining isolates (n = 38) were randomly collected from different wards of the hospital during the same period, so as to further investigate the extent and the epidemiology of the outbreak.

During September to December 2012, repeated surveys of rectal and pharyngeal swabs were obtained from neonates on a weekly basis. Screening of nursing staff and environmental contamination by ESBL-Kp was also carried out. All aforementioned surveillance cultures (1258 cultures in total) were directly inoculated on MacConkey agar plates. Recovered ESBL-Kp organisms were stored at -80°C in Trypticase Soy Broth containing 10% (v/v) glycerol for further analysis.

### Detection of beta-lactamase and virulence genes

Total DNA from all ESBL-Kp was extracted using the Quick-gDNA TM MiniPrep kit (ZYMO RESEARCH Corp., USA). Detection by PCR of beta-lactamase (*bla*) genes encoding KPC-, VIM-, TEM-, OXA- and CTX-M-type enzymes was performed, as described previously [14]. The intrinsic SHV-1 and SHV-5-type enzymes were differentiated by PCR, as described previously [15]. Sequences of the PCR products were determined in both strands. Production of the respective beta-lactamases was confirmed by isoelectric focusing (IEF) [14]. The presence of the *fimH*, *ugeE*, *wabG, ureA*, *magA*, *allS* and *rmpA* virulence genes was assessed by PCR, as described previously [16].

### Genotyping and phylogenetic analysis

Molecular typing was performed by BOX-PCR and Multilocus Sequence Typing (MLST), as described previously [17,18]. Allele numbers and sequence types (STs) were assigned and new STs were deposited on the Institut Pasteur France *K. pneumoniae* MLST database (http://www.pasteur.fr/mlst). Phylogenetic analysis was performed by the neighbor-joining tree algorithm using the MEGA software [19].

### Collection of clinical data

Before obtaining the clinical information of the neonates, approval was received by the Ethics Committee of the UHL, which is represented by the Infection Control Committee (number of permission 1234). Clinical records from the neonates were collected and reviewed including the following data: date and place of birth, date of admission in the NICU, length of stay in the NICU, birth weight, sex, vaginal or caesarean delivery, gestational age, age (days after birth) at first isolation of ESBL-Kp, surgical procedures, intubation, use of central venous catheters, placement of chest tubes, parenteral nutrition, antimicrobial therapy and use of intralipids.

## Results and discussion

### Outbreak description and infection control interventions

The onset of the outbreak was recognised on the 5^th^ March 2012 when two ESBL-Kp isolates were recovered from the blood and urine samples of two twin neonates born at the maternity unit of the UHL. These isolates were recovered on the 13^th^ and 18^th^ day after admission of the twins in the NICU, suggesting acquisition of these isolates in the NICU. Another case was detected on the 30^th^ March and two more cases at the beginning of April.

On the 15th April, a special team (one microbiologist and two nurses) was formed to coordinate the management of the outbreak, providing specific recommendations, such as cohorting of infants, limited rotation of the staff, encouraging effective hand hygiene, safe disposal of diapers into specific bags, daily cleaning of the surfaces and soiled articles with soap and water, followed by disinfection with a dilute solution of chlorine containing bleach and chlorhexidine (Acrylan, Kosmidis Company, Athens, Greece). No new cases were detected between May and June, and despite the control measures, three new cases were detected from July to August 2012. During this period, we noted that the ratio of nurse/patients was 1:7 at the time of the outbreak because of understaffing because of summer leaves (usual ratio, 1:4). Thus, from March to August 2012, a total of 8 ESBL-Kp were detected in an equal number of neonates.

On the 1^st^ September 2012, a surveillance protocol was implemented for all neonates affected by ESBL-Kp in a weekly basis. From September to December 2012, five more infected neonates were identified, whereas 13 ESBL-Kp were recovered from 12 colonized infants. Four ESBL-Kp were recovered from blood cultures within the first three weeks of November, and therefore infection control measures were intensified. On the 1st December, three cohorts of infants were established: the first group included all infants infected or colonized with ESBL-Kp which were cared for by designated nurses and placed on contact precautions until hospital discharge in a separate nursery, the second group included infants with exposure to case-infants, but with negative surveillance cultures, which were cared for by another group of designated nurses, and a third group of newly admitted infants were cared for in a separate room by another group of designated nurses. Furthermore, the antibiotic policy in the NICU has changed; restriction of third-generation cephalosporins was enforced and imipenem was used for infants with suspected sepsis. Multidisciplinary meetings were held twice weekly to discuss the ongoing investigation and compliance with the infection control measures. From September to December 2012, a total of 18 ESBL-Kp were detected in 5 infected and 12 colonized neonates.

ESBL- Kp have not been isolated from the hands of the nursing staff and environmental samples. No other cases were detected until now and we assumed that the outbreak was successfully controlled.

### Characterization of beta-lactamase and virulence gene content

All 26 neonatal ESBL-Kp were positive only for both the SHV-5 and TEM-1 beta-lactamases, as shown by PCR and IEF. Among the 38 ESBL-Kp collected from other wards of the hospital, 18 isolates were SHV-12 (n = 12) or SHV-5 (n = 6) producers, 19 isolates were CTX-M-15 (n = 16) or CTX-M-3 (n = 3) producers and one isolate coproduced SHV-5 and CTX-M-15. All neonatal ESBL-Kp were also positive for the presence of *fimH*, *ugeE*, *wabG* and *ureA*, but negative for the *magA*, *allS* and *rmpA* virulence genes.

### Antimicrobial susceptibility testing and molecular typing

The 64 ESBL-Kp were assigned to 15 different BOX-PCR profiles. Among 32 representative isolates of the major BOX-PCR profiles and all the isolates with unique profiles, we have identified 13 MLST STs; five of them were novel STs. The antimicrobial susceptibility patterns of the isolates and the STs of the four major BOX-PCR profiles identified among the 64 ESBL-Kp are shown in Table 1.

**Table 1:** Antimicrobial susceptibility patterns of the four major BOX-PCR profiles of ESBL-Kp in a NICU, Larissa, Greece

**BOX-PCR profile**^***a***^	**MLSTST**					**MIC range (mg/L)**^***b***^							
		**AMP**	**AMP/SUL**	**CTX**	**CAZ**	**ATM**	**IMP**	**MER**	**ERT**	**CIP**	**TOB**	**GM**	**TSX**
**NICU (n = 26)**													
P1 (16)	ST20	≥32	≤2- 16	≤1- ≥64	8- ≥64	8- ≥64	≤1	≤0.25	≤0.5	≤0.25	≥16	≥16	≤20
P2 (8)	ST1114	≥32	≤2	≤2- ≥64	16- ≥64	4- ≥64	≤1	≤0.25	≤0.5	≤0.25	≥16	≥16	≤20
**Non-NICU (n = 38)**													
P5 (12)	ST258	≥32	8- ≥32	16	≥64	≥64	≤1	≤0.25	≤0.5	≥4	≤1-16	≥16	≤20- ≥320
P6 (16)	ST101	≥32	≥32	≥64	16- ≥64	32- ≥64	≤1	≤0.25	≤0.5	≥4	8- ≥16	≤1-8	≤20- ≥320

^*a*^In parenthesis, the number of isolates identified in each of the four major BOX-PCR profiles is shown.

^*b*^AMP: ampicillin, AMP/SUL: ampicillin/sulbactam, ATM: aztreonam, CAZ: ceftazidime, CIP: ciprofloxacin, CTX: cefotaxime, ERT: ertapenem, GM: gentamycin, IMP: imipenem, MER: meropenem, TOB: tobramycin, TSX: trimethoprim/sulfamethoxazol.

**Figure 1:**
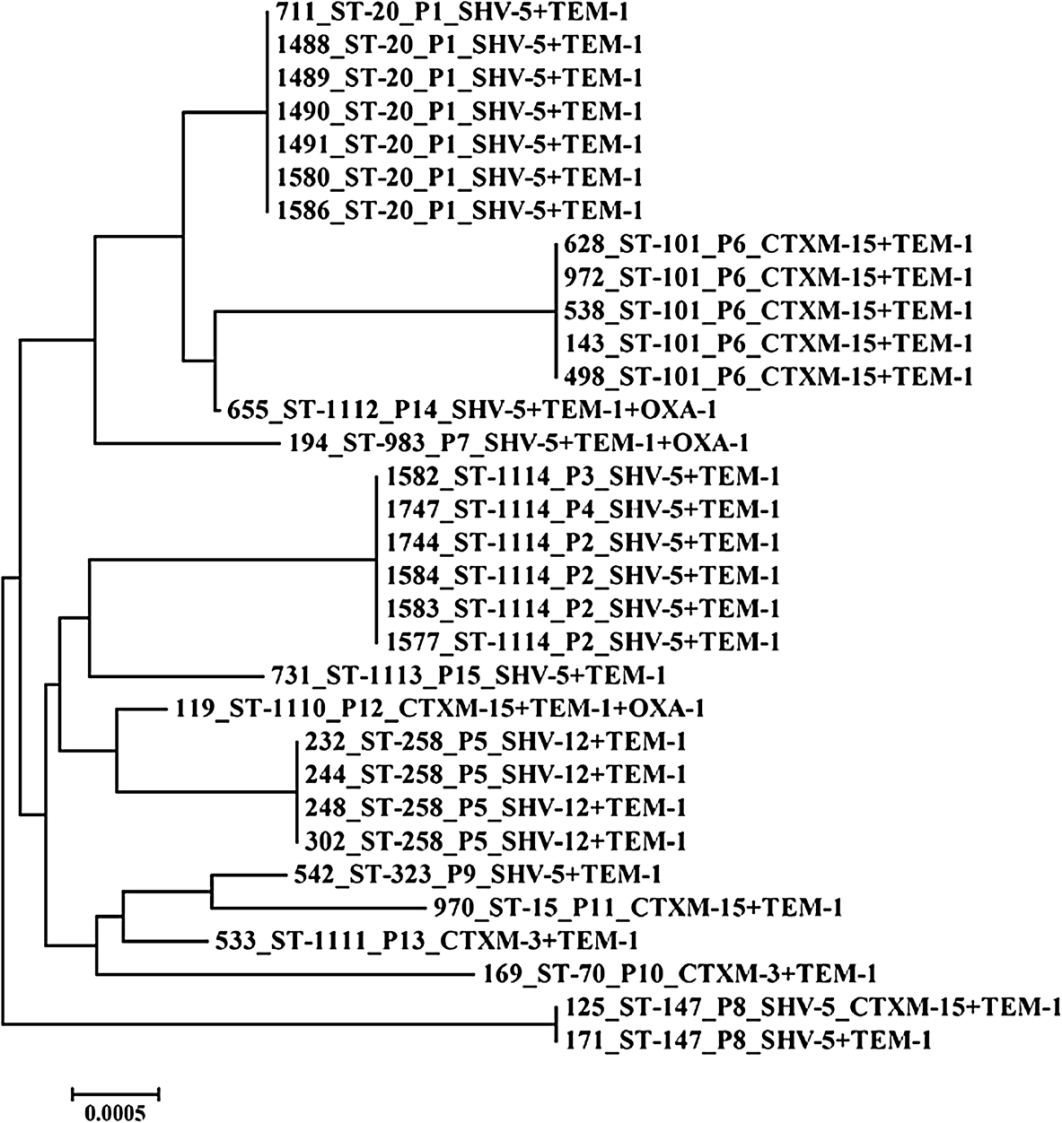
**Neighbor-joining tree of the STs of the 32 representative ESBL-Kp.** For each isolate, the MLST ST, the BOX-PCR profile and the beta-lactamase content are indicated.

All neonatal ESBL-Kp displayed multidrug-resistance phenotypes, including resistance to penicillins, ceftazidime, and aztreonam, tobramycin and gentamycin (Table 1). ST20 (BOX-PCR pattern P1) was identified among 16 ESBL-Kp recovered from 13 infected and three colonized neonates. ST20 has been previously identified among newborns affected by ESBL-Kp, which produced CTXM-15 in Spain [20]. A novel ST (ST1114) was identified in nine neonates colonized by ESBL-Kp (n = 10) of the BOX-PCR patterns P2 (n = 8), P3 (n = 1) and P4 (n = 1) (Figure 1). The 38 ESBL-Kp recovered from the other wards of UHL were distributed into two major clusters, P5 (n = 12) and P6 (n = 16) profiles (Table 1, Figure 1), which were assigned to ST258 and ST101, respectively. ST258 has been identified mostly among carbapenemase-producing *K. pneumoniae* worldwide (including Greece), whereas ST101 has been previously reported among carbapenemase-producing *K. pneumoniae* in Italy and Korea [3,21-23].

Neonatal ESBL-Kp of ST20 and ST1114 were not genetically related, each comprising a separate lineage on the neighbor-joining tree (Figure 1). Furthermore, ST20 and ST1114 were not found among non-carbapenemase-producing ESBL-Kp recovered from other wards of the hospital. Although SHV-5 producers were distributed into six different MLST STs, there was no genetic relatedness among the neonatal and the other strains (Figure 1). Therefore, transmission of SHV-5 producers from the NICU to other wards, or vice versa, was not documented.

The differences in the antibiotic resistance profiles of the neonatal ESBL-Kp with those recovered from other wards of the hospital (Table 1) and the presence of two distinct bacterial clones among ESBL-Kp in the NICU, the ST20 and ST1114 clones (corresponding to BOX-PCR profiles P1 and P2, respectively), which were not genetically related to each other or other ESBL-Kp isolated from other wards of the hospital (Table 1, Figure 1), indicate that ESBL-Kp should have been imported in two different occasions and disseminated only in the NICU.

**Figure 2:**
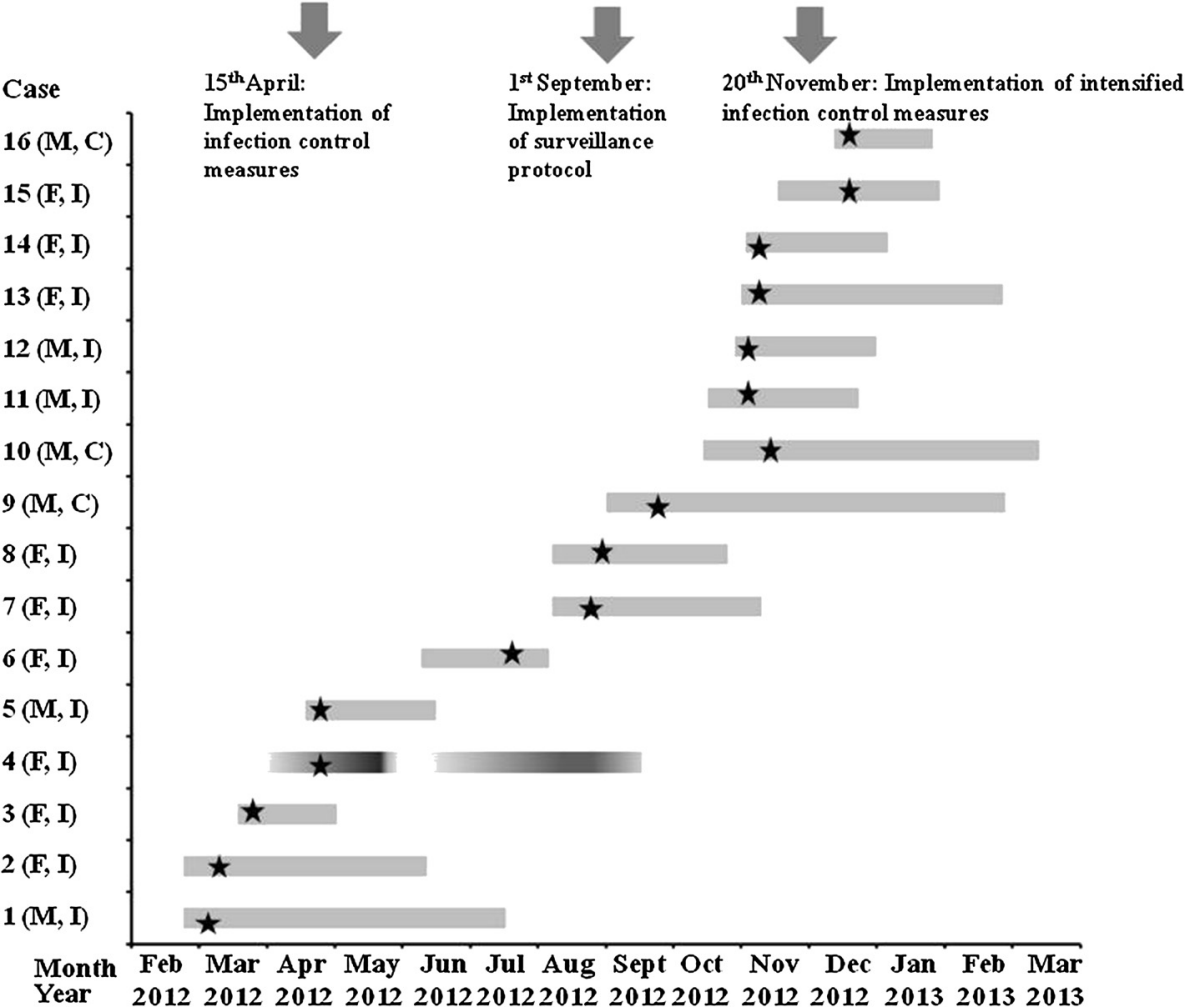
**Time-course of the outbreak of ST20 ESBL-Kp.** The sex (F: female, M: male) and the infection (I) or colonization (C) status of each infant is indicated. Case 4, indicated with a different colour scheme, has died. The time of first isolation of ST20 SHV-5 producers for each infant is indicated with an asterisk.

As all of the infected neonates and only three colonized neonates were affected by ST20 ESBL-Kp, we considered that this clone was the main cause of the outbreak in the NICU. The time-course of the outbreak of ST20 ESBL-Kp is shown in Figure 2. ESBL-Kp of ST1114 were identified in the NICU from September to December 2012, when surveillance cultures were obtained from the neonates. However, we cannot exclude the possibility that the ST1114 ESBL-Kp were circulating in the NICU among colonized infants before the surveillance period. Nevertheless, we were not able to identify the source of the importation of ESBL-Kp in the NICU, as no ESBL-Kp were isolated either from the hands of the nursing staff or the environment. It could be hypothesized that some of the mothers of the neonates were carriers of ESBL-Kp and that they were the source of the neonatal infections or colonizations. However, screening for colonization of the mothers of the neonates was not performed in this study.

### Clinical characteristics of the outbreak

A retrospective review of clinical data of the neonates affected ESBL-Kp was performed (Table 2). Of the 16 (13 infected and three colonized) neonates affected by the outbreak strain of ST20 ESBL-Kp, 11 neonates were born in the maternity unit of UHL, whereas the remaining neonates were admitted to UHL from the general hospital of Larissa or different private maternity clinics of the prefecture of Thessaly. One infected neonate has died. Fourteen of the 16 neonates were delivered by caesarean section and they showed low gestational age (≤32 weeks) and low birth weight (≤1500 g). All neonates had received parenteral nutrition. Several risk factors, such as intubation (n = 13), placement of central venous catheters (n = 11) and chest tubes (n = 4) were also documented. Low gestational age, low birth weight and use of invasive devices have been reported previously among the risk factors for acquiring ESBL-producing Enterobacteriaceae in NICUs [6,24-27].

**Table 2:** Clinical characteristics of the neonates affected by ESBL-Kp in a NICU, Larissa, Greece

**Cases**	**Birth date**	**Admission date**	**Gestational age at birth (weeks)**	**Age at first isolation of ESBL-Kp (days)**	**Length of stay (days)**	**Weight at birth (g)**	**Gender**^***a***^	**Inborn/Outborn**	**Source**^***b***^	**Infected/colonized**	**Delivery**^***c***^	**Risk factors**^***d***^	**Outcome**
***Neonates affected by ST20 ESBL-Kp***													
1	February 23	February 23	27	10	120	1000	M	Inborn	ET	Infected	CS	PN, IT,CVC	Discharged
2	February 23	February 23	27	10	85	1070	F	Inborn	Urine	Infected	CS	PN, IT,CVC	Discharged
3	March 17	March 17	31	13	21	1220	F	Inborn	Urine	Infected	CS	PN	Discharged
4	March 28	March 28	26	34	145	790	F	Outborn	ET	Infected	CS	PN, IT, CVC	Died
5	April 19	April 20	38	4	35	3380	M	Outborn	ET	Infected	CS	PN, IT, CVC, CT	Discharged
6	July 8	July 8	32	11	34	1360	F	Outborn	Eye	Infected	CS	PN, IT,	Discharged
7	August 6	August 6	28	3	70	1255	F	Inborn	Catheter	Infected	CS	PN, IT, CVC, CT	Discharged
8	August 6	August 6	28	11	55	1000	F	Inborn	ET	Infected	CS	PN, IT, ET, CVC	Discharged
9	August 31	August 31	28	22	154	800	M	Inborn	FC	Colonized	CS	PN, IT, CVC	Discharged
10	October 13	October 13	28	35	126	960	M	Inborn	FC	Colonized	CS	PN, IT, CVC, CT	Discharged
11	October 15	October 15	31	25	44	1500	M	Inborn	Blood	Infected	VD	PN, IT, CVC	Discharged
12	October 27	October 27	31	6	40	580	M	Outborn	Blood	Infected	CS	PN, IT, CVC	Discharged
13	October 31	October 31	28	20	93	1120	F	Outborn	Blood	Infected	CS	PN, IT, CVC	Discharged
14	November 2	November 2	30	7	40	1350	F	Outborn	Blood	Infected	CS	PN	Discharged
15	November 16	November 16	32	32	49	1365	F	Inborn	Urine	Infected	VD	PN, IT	Discharged
16	December 6	December 09	35	10	21	2270	M	Outborn	FC	Colonized	CS	PN	Discharged
			Range: 26–38	Range: 3–35	Range: 21–154	Range: 580–3380							
			Mean: 30	Mean: 15.8	Mean: 70.8	Mean: 1313.8							
***Neonates affected by ST1114 ESBL-Kp***													
17	September 5	September 5	28	25	35	850	F	Inborn	FC	Colonized	CS	PN	Discharged
18	September 15	September 15	27	17	80	1000	M	Outborn	FC	Colonized	CS	PN, IT, CVC	Discharged
19	October 5	October 5	30	15	45	750	F	Inborn	FC	Colonized	CS	PN	Discharged
20	October 27	October 27	31	25	56	1000	F	Inborn	FC	Colonized	CS	PN,CVC	Discharged
21	October 31	October 31	28	30	118	1040	F	Outborn	FC	Colonized	CS	PN, IT,CVC	Discharged
22	November 5	November5	28	12	114	980	F	Outborn	FC	Colonized	VD	PN, IT,CVC	Discharged
23	November 15	November 15	27	35	90	1070	F	Inborn	FC	Colonized	VD	PN, IT,CVC	Discharged
24	December 22	December22	34	4	27	1480	F	Outborn	FC	Colonized	CS	PN	Discharged
25	December 28	December 28	31	2	27	1480	F	Outborn	FC	Colonized	CS	PN	Discharged
			Range: 27–34	Range: 2–35	Range: 27–118	Range: 750–1480							
			Mean: 29.3	Mean: 18.3	Mean: 65.7	Mean: 1072.2							

^*a*^F: female, M: male.

^*b*^ET: endotracheal tube, FC: Fecal carriage.

^*c*^CS: caesarean section, VD: vaginal delivery.

^*d*^CT: chest tubes, CVC: central venous catheters, IT: intubation, PN: parenteral nutrition.

As mentioned previously, of the 25 neonates affected by ESBL-Kp and 16 neonates affected by ST20 ESBL-Kp, 13 (52% and 81.2%, respectively) developed an infection, whereas ST1114 was identified only among colonized patients (Table 2). Thus, ST20 ESBL-Kp showed high infectiousness compared with ST1114 ESBL-Kp. Furthermore, four out of 16 (25%) ST20 ESBL-Kp were recovered from bloodstream infections. These observations could be attributed to differences either in the virulence potential of the STs or in the clinical characteristics of the infants affected by ST20 and ST1114 ESBL-Kp. No differences in the virulence gene content among ESBL-Kp belonging to ST20 and ST1114 were observed, but several other virulence characteristics (e.g. the mucoid phenotype, aerobactin production, capsular serotype) are associated with the type of infection in *K. pneumoniae*, as reported previously [28]. As shown in Table 2, no differences in the clinical characteristics of the neonates affected by ST20 and ST1114 ESBL-Kp were observed.

In our study, surveillance cultures were obtained during the outbreak period (from September- December 2012). The percentage of new carriers out of the total amount that were screened during the out break period (12 colonized infants out of 62 infants screened from September to December 2012) was 19.35%. Surveillance has stopped by the end of December of 2012, as no new infections by ESBL-Kp were detected after the end of December 2012 and onwards, only a small percentage of ST20 ESBL-Kp were detected in colonized infants (3 out of 16 ST20 ESBL-Kp) during the outbreak period, and no infections were caused by ST1114 ESBL-Kp. The best time to screen neonates is arguable and high compliance with the surveillance protocol is essential for success. Nevertheless, it has been shown recently that continuous long-term surveillance and neonatal cohorting are associated with a marked decrease in the spread of ESBL-KP within the NICU [27].

## Conclusions

In the present study, we described an outbreak occurred during a nine-months period in 2012 at the NICU of UHL caused mainly by multidrug-resistant SHV-5 producers of ST20 recovered from 13 infected and three colonized neonates, whereas a novel ST (ST1114) was identified only among colonized neonates. A retrospective study of the clinical data of the neonates affected by ESBL-Kp has revealed that all neonates had received parenteral nutrition and most of them were delivered by caesarean section and showed low birth weights. To our knowledge, this is the first report of SHV-5 producers assigned to ST20. The emergence of ST20 and ST1114 SHV-5 producers in the NICU, along with the presence of various previously reported STs (e.g. ST101, ST258) and six novel STs among SHV-5/12 or CTXM-3/15 producers in other wards of UHL indicate the ongoing evolution of ESBL-producing *K. pneumoniae* in our area.

## Abbreviations

DDST: Double-disk synergy test; ESBL: Extended-spectrum beta-lactamase; ESBL-Kp: Extended-spectrum beta-lactamase-producing *Klebsiella pneumoniae*; ICU: Intensive care unit; MLST: Multilocus sequence typing; NICU: Neonatal intensive care unit; UHL: University hospital of Larissa.

## Competing interests

The authors declare that they have no competing interests.

## Authors’ contributions

EP, AM, and VM conceived and designed the study. AM wrote the first draft of the paper and other co-authors contributed to the final draft. AL and AM performed the experiments. AG, MG and KG were responsible for managing the clinical data. EP, AG, AM and VM conducted the interpretation of data. All authors read and approved the final manuscript.

## Pre-publication history

The pre-publication history for this paper can be accessed here:

http://www.biomedcentral.com/1471-2431/14/105/prepub
